# Supplementary material for: A maximum-entropy model to predict 3D structural ensembles of chromatin from pairwise distances with applications to interphase chromosomes and structural variants
Source: Nat Commun. 2023 Mar 1;14:1150. doi: 10.1038/s41467-023-36412-4 (PMC9974990; doi:10.1038/s41467-023-36412-4)
Supplement: Supplementary file 1 — Supplementary Information [file 41467_2023_36412_MOESM1_ESM.pdf]

Supplementary Information: A maximum-entropy method to predict 3D structural ensembles of chromatins from pairwise distances with applications to Interphase Chromosomes and Structural Variants

Guang Shi[1,2,*] and D. Thirumalai[1,3,*]

[1] *Department of Chemistry, University of Texas at Austin, Austin, Texas 78712, USA*

[2] *Current Address: Department of Materials Science, University of Illinois, Urbana, Illinois 61801, USA*

[3] *Department of Physics, University of Texas at Austin, Austin, Texas 78712, USA*

SUPPLEMENTARY NOTE 1 – ALGORITHMS

The objective in the application of the maximum entropy principle is to find a distribution that maximizes the entropy among all the distributions, which also satisfies a set of specified constraints. In a typical application, the constraints are the m mean values, which are functions over certain random variables. We write these constraints as $\mathbb{E}[f_i(\boldsymbol{x})] = \hat{a}_i$ where $f_i(\boldsymbol{x})$ is the ith function over the random variable $\boldsymbol{x}$. The maximum entropy distribution is given by the following form,

$$P^{\text{MaxEnt}}(\boldsymbol{x}) = \frac{1}{Z}\exp\big(\sum_i^m \theta_i f_i(\boldsymbol{x})\big) \tag{1}$$

where θ_i are the Lagrange multipliers that have to be determined. The values of θ_i are determined so that the constraints are satisfied, that is,

$$\int \mathrm{d}\boldsymbol{x} P^{\text{MaxEnt}}(\boldsymbol{x}) f_i(\boldsymbol{x}) = \hat{a}_i, \text{ for all } i \tag{2}$$

According to [1, 2], the problem of finding the values of θ_i is equivalent to minimizing the objective function,

$$L(\boldsymbol{\theta}) = \ln Z(\boldsymbol{\theta}) - \boldsymbol{\theta}^T \hat{\boldsymbol{a}} \tag{3}$$

where $\boldsymbol{\theta} = [\theta_1, \theta_2, \cdots, \theta_m]$, $\hat{\boldsymbol{a}} = [\hat{a}_1, \hat{a}_2, \cdots, \hat{a}_m]^T$, and $Z(\boldsymbol{\theta}) = \int \mathrm{d}\boldsymbol{x} \, \exp\big(\sum_i^m \theta_i f_i(\boldsymbol{x})\big)$ is the normalization factor (partition function). It can be shown that $L(\boldsymbol{\theta})$ is a global minimum when all the constraints $\mathbb{E}[f_i(\boldsymbol{x})] = \hat{a}_i$ are satisfied. Hence, the problem of finding the maximum entropy distribution is equivalent to optimizing $L(\boldsymbol{\theta})$. We now describe two methods for solving the optimization problem.

1. Iterative scaling

Iterative scaling [3] is one of the earliest methods developed for numerically solving the maximum entropy problem. The updating scheme for $\boldsymbol{\theta}$ is the following,

$$\boldsymbol{\theta}_{t+1} = \boldsymbol{\theta}_t + \frac{\gamma}{\sum_i \langle f_i \rangle_{\boldsymbol{\theta}_t}} \ln \frac{\langle \boldsymbol{f} \rangle_{\boldsymbol{\theta}_t}}{\hat{\boldsymbol{a}}} \tag{4}$$

* Corresponding author emails: guang.shi.gs@gmail.com; dave.thirumalai@gmail.com

where $\langle \boldsymbol{f} \rangle_{\boldsymbol{\theta}_t}$ is the vector associated with the constraint functions $f_1, f_2, \cdots, f_m$ evaluated at the current $\theta_1, \theta_2, \cdots, \theta_m$, γ is the constant learning rate.

2. Gradient descent (GD)

A typical procedure to implement the GD method involves solving,

$$\boldsymbol{\theta}_{t+1} = \boldsymbol{\theta}_t - \gamma_t \nabla L(\boldsymbol{\theta}_t) \tag{5}$$

where the step size (learning rate) is either a constant ($\gamma_t = \gamma$) or allowed to change at each step, t. The gradient of $L(\boldsymbol{\theta})$ is,

$$\frac{\partial L(\boldsymbol{\theta})}{\partial \theta_i} = \langle f_i \rangle_{\boldsymbol{\theta}} - \hat{a}_i \tag{6}$$

where the subscript $\boldsymbol{\theta}$ indicates the average is evaluated at the parameters $\boldsymbol{\theta}$.

3. Regularization

For Chromosome 2, investigated in the main text, the positions of $\sim$900 loci were measured, resulting in $\approx 900 \times 900/2 \approx 4 \times 10^5$ parameters (number of k_{ij} values) that need to be determined. With this many parameters, regularization is usually needed in order to avoid overfitting. In this work, unless specified otherwise, we employ the L_2 regularization, which adds a penalty term to the objective function,

$$\tilde{L}(\boldsymbol{\theta}) = \ln Z(\boldsymbol{\theta}) - \boldsymbol{\theta}^T \hat{\boldsymbol{a}} + \lambda ||\boldsymbol{\theta}||_2^2. \tag{7}$$

The penalty term $\lambda ||\boldsymbol{\theta}||_2^2$ constrains the values of θ_i to be close to zero. The parameter λ controls the contribution of the penalty term. To minimize $\tilde{L}(\boldsymbol{\theta})$, we employ the gradient descent method. The gradient of $\tilde{L}(\boldsymbol{\theta})$ is given by,

$$\frac{\partial \tilde{L}(\boldsymbol{\theta})}{\partial \theta_i} = \langle f_i \rangle_{\boldsymbol{\theta}} - \hat{a}_i + 2\lambda \theta_i. \tag{8}$$

SUPPLEMENTARY NOTE 2 – CORRELATION MATRIX

In this note, we describe how to calculate the correlation matrix given the connectivity matrix $\boldsymbol{K}$. Let us denote the correlation matrix as $\boldsymbol{\rho}$. The elements of $\boldsymbol{\rho}$, ρ_{ij}, are defined as,

$$\rho_{ij} = r_s(\boldsymbol{K}_i, \boldsymbol{K}_j) \tag{9}$$

where $\boldsymbol{K}_i$ and $\boldsymbol{K}_j$ are the ith row and j^{th} row of matrix $\boldsymbol{K}$, respectively, $r_s(\cdot)$ is the standard function used to compute the Spearman's rank correlation coefficient between $\boldsymbol{K}_i$ and $\boldsymbol{K}_j$. The calculation is done using *SciPy* Python package [4]

SUPPLEMENTARY NOTE 3 – STRUCTURAL VARIATIONS

In this note, we describe how the connectivity matrix $\boldsymbol{K}$ is modified in modeling of structural variations (shown schematically in Supplementary Fig.5), which are mutations in the chromosomes. Our goal is to utilize the calculated $\boldsymbol{K}$ for the wild-type chromosome, and then predict the structural changes in the chromosome *without* any additional inputs or modifications.

Inversion

Inversion means that a genome sequence is reversed from end-to-end. Let the inverted segment lie between m^{th} and n^{th} loci ($m < n$) in the wild type (WT). We account for the effects of inversion is by modifying only the elements of $\boldsymbol{K}$ associated with the loci involved in the inversion process. More precisely, the modification of $\boldsymbol{K}$ can be summarized as,

$$\begin{aligned}
K_{ij}^{\mathrm{INV}} &= K_{ij}^{\mathrm{WT}}, \text{ if } i \not\subset [m,n] \text{ and } j \not\subset [m,n] \\
K_{ij}^{\mathrm{INV}} &= K_{i,m+n-j}^{\mathrm{WT}}, \text{ if } i < m \text{ and } j \subset [m,n] \\
K_{ij}^{\mathrm{INV}} &= K_{m+n-i,j}^{\mathrm{WT}}, \text{ if } j > n \text{ and } i \subset [m,n] \\
K_{ij}^{\mathrm{INV}} &= K_{m+n-i,m+n-j}^{\mathrm{WT}}, \text{ if } i \subset [m,n] \text{ and } j \subset [m,n]
\end{aligned}$$

where the superscripts denote inversion (INV) and wild type (WT), respectively.

Deletion

Deletion corresponds to a deletion of a genome sequence, which would shorten the number of base pairs. As before, let the segment deleted be located between m^{th} and n^{th} loci ($m < n$) in the wild type. The deletion effect is modeled by removing the k_{ij} elements from $\boldsymbol{K}$ involving the deleted sequence. The modification of $\boldsymbol{K}$ is implemented using,

$$
\begin{aligned}
K_{ij}^{\mathrm{DEL}} &= K_{ij}^{\mathrm{WT}}, \text{ if } i,j < m \text{ or } i,j > m \\
K_{ij}^{\mathrm{DEL}} &= K_{i,n+j-m}^{\mathrm{WT}}, \text{ if } i < m \text{ and } j > m \\
K_{ij}^{\mathrm{DEL}} &= (K_{m-1,m}^{\mathrm{WT}} + K_{m,m+1}^{\mathrm{WT}})/2, \text{ if } i = m-1 \text{ and } j = m
\end{aligned}
$$

where the superscript DEL denotes deletion.

In both inversion and deletion variants, we set $K_{ij} = K_{ji}$. The diagonal elements in $\boldsymbol{K}$ is computed using $K_{ii} = -\sum_{j \neq i} K_{ij}$. It is worth emphasizing that in determining the effects of inversion and deletion the $\boldsymbol{K}$ matrix is determined only once for the wild type.

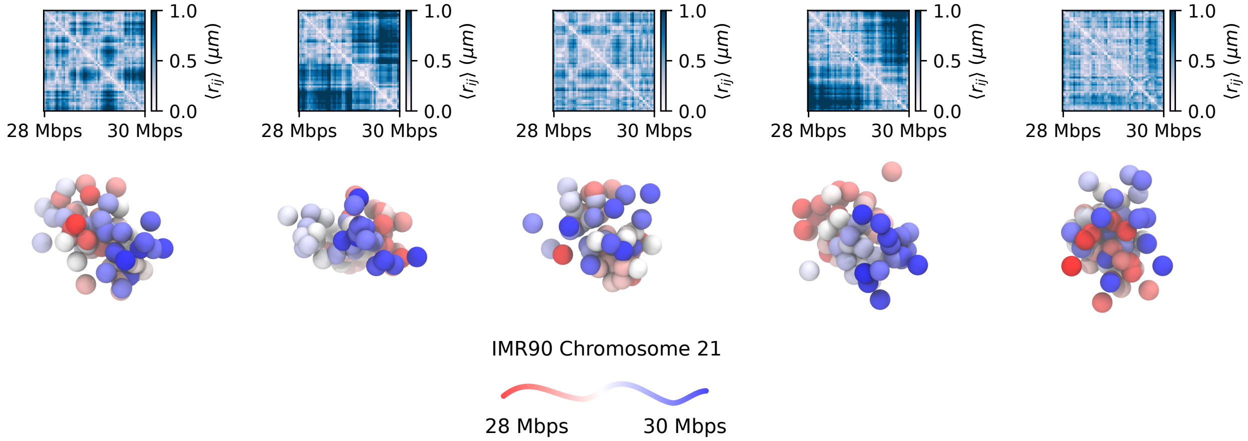

FIG. 1. Examples of the distance map for a few individual conformations for Chr21 in the 28 Mbps - 30 Mbps region in IMR90 cell line. Each panel shows an individual structure randomly chosen from the ensemble from which the spatial distance matrix is computed. The distance scale is given on the right of the panels. Just as in experiments [5], TAD-like or domain-like patterns are clearly observed in the spatial distance matrices even at the single cell level. The snapshots below show the structures. Each bead in the structure represents a single locus. The color of the loci in the 3D structures represents the genomic location, ranging from red to blue.

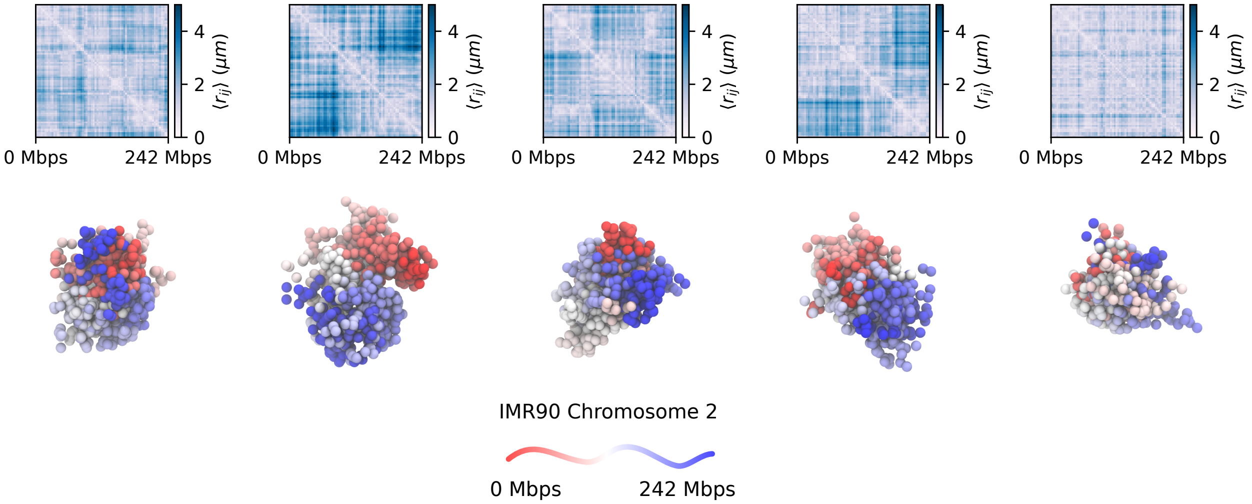

FIG. 2. Distance maps (scales on the right) for individual conformations for the nearly 242 Mbps Chr2 in IMR90 cell line. We find substantial variations in the distance maps in individual conformations, attesting to the heterogenity in the genome organization. This observation accords well with experiments [6]. In the snap shots below, each bead represents one locus. The color of loci in the 3D structures represents the its genomic location, ranging from red to blue.

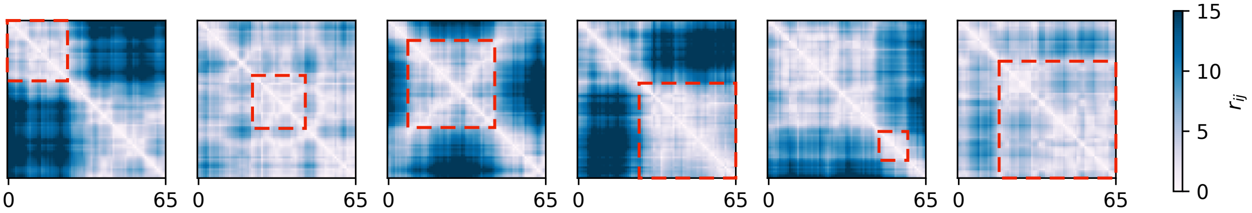

FIG. 3. Distance maps (scales on the right) for random sampled conformations for an ideal Rouse homopolymer (the number of monomers is 65). We find substantial variations in the distance maps in individual conformations, which stem from the intrinsic thermal fluctuations of the ensemble. TAD-like domains are discernible in the distance matrices. A few examples of such TAD-like structures are marked by dashed squares.

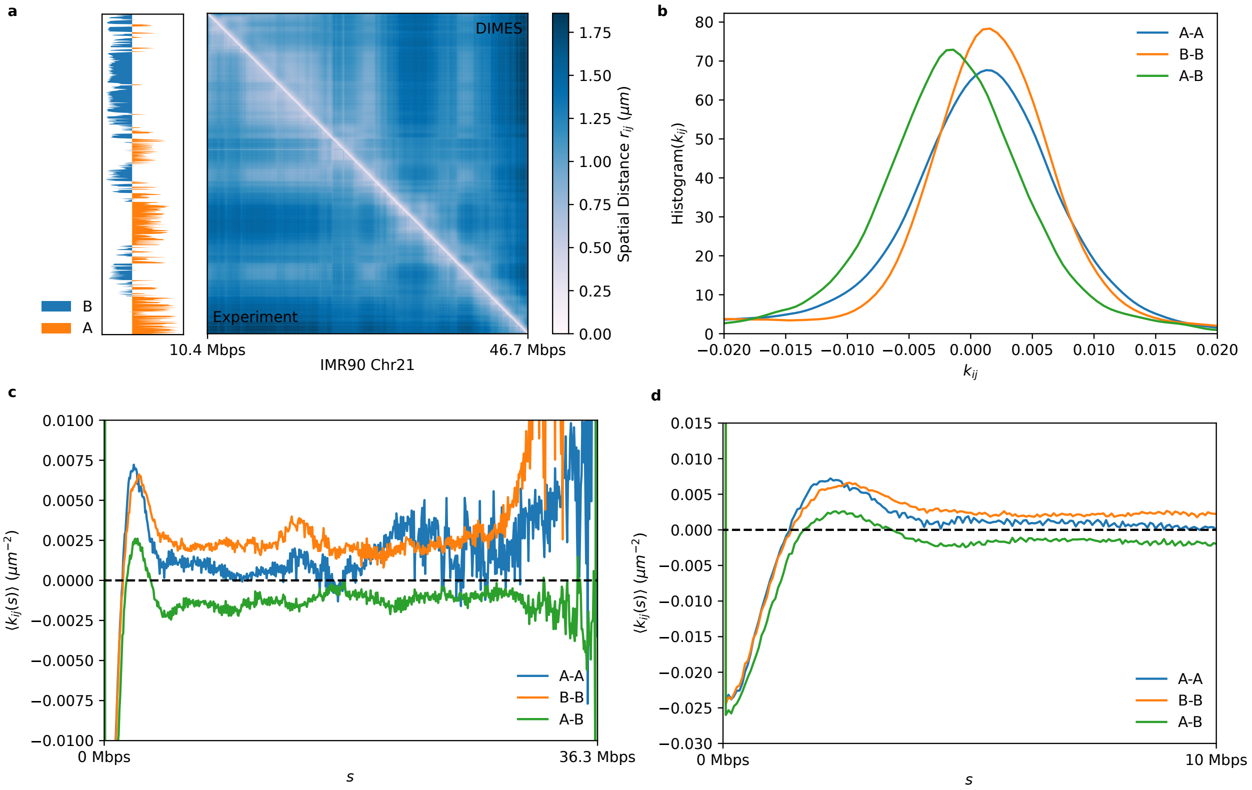

FIG. 4. Characteristics of pair-wise interactions of the Chromosome 21 for IMR90 cell line. **(a)** The average distance matrix between experiment (lower triangle) and the DIMES (upper triangle) show excellent agreement. The left track shows the principle component dimension 1 (PC1) computed using principle component analysis (PCA) from the connectivity matrix $\boldsymbol{K}$. **(b)** Histogram of k_{ij} for A-A, B-B and A-B. **(c)** Genomic-distance normalized $\langle k_{ij}(s)\rangle = (1/(N-s)) \sum_{i<j}^{N} \delta(s-(j-i))k_{ij}$ for A-A, B-B and A-B. $\langle k_{ij}(s)\rangle$ are shown for s between 0 and 36.3 Mbps. **(d)** Enlarge portion of **(c)**, showing $\langle k_{ij}(s)\rangle$ for $s < 10$ Mbps.

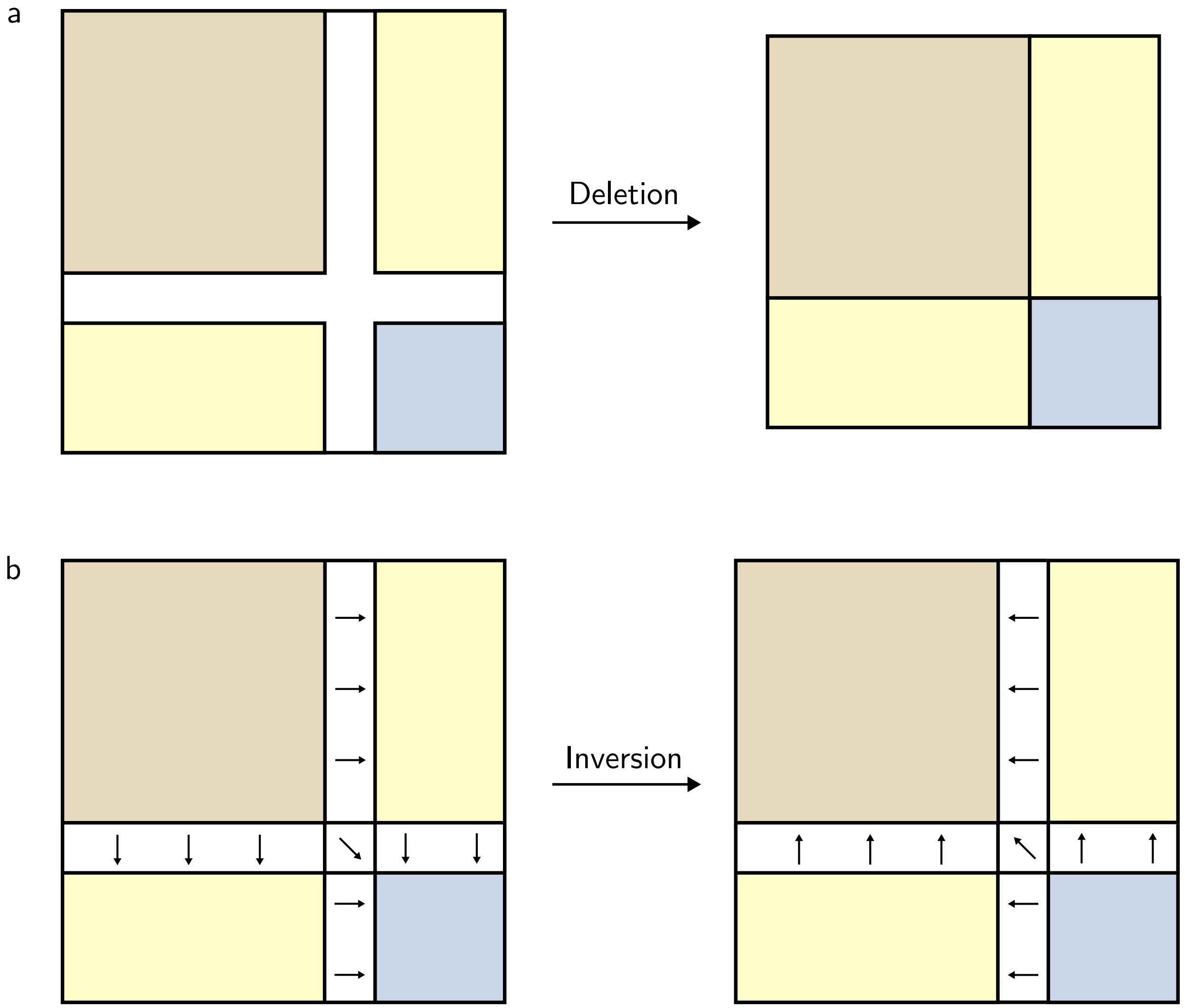

FIG. 5. Schematics for deletion operation and inversion operation on connectivity matrix $\boldsymbol{K}$. **(a)** Illustration of deletion. Different parts of the matrix $\boldsymbol{K}$ are in different colors. The gray area corresponds to the region deleted. The resulting $\boldsymbol{K}$ is shown on the right. **(b)** The gray areas with gradient correspond to the inverted segment. The change of direction of gradient after the inversion depict how the values of elements in $\boldsymbol{K}$ are altered.

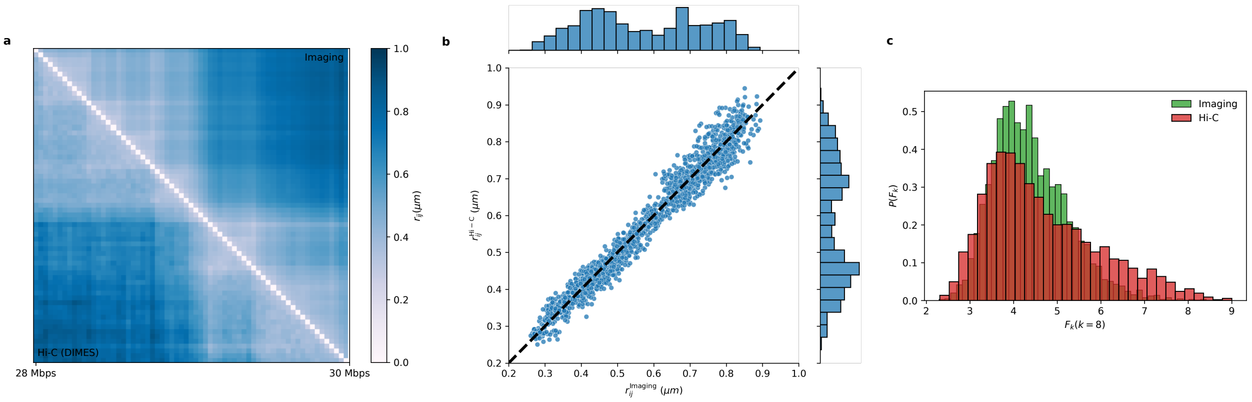

FIG. 6. Structural organization calculated from Hi-C and imaging data for Chr21 28 Mbps - 30 Mbps from IMR90 cell line. **(a)** Comparison between the mean distance matrix inferred from Hi-C contact map (lower triangle) and the experimental measured average distance matrix (upper triangle). The distance scale is shown on the right. **(b)** Direct comparison of pairwise distances, $\langle r_{ij}^{\text{Imaging}} \rangle$ versus $\langle r_{ij}^{\text{Hi-C}} \rangle$. Each dot represents a pair (i, j). Dashed line, with a slope of unity, is a guide to the eye. **(c)**. Histogram of $F_k(k = 8)$ for 4,000 individual structures.

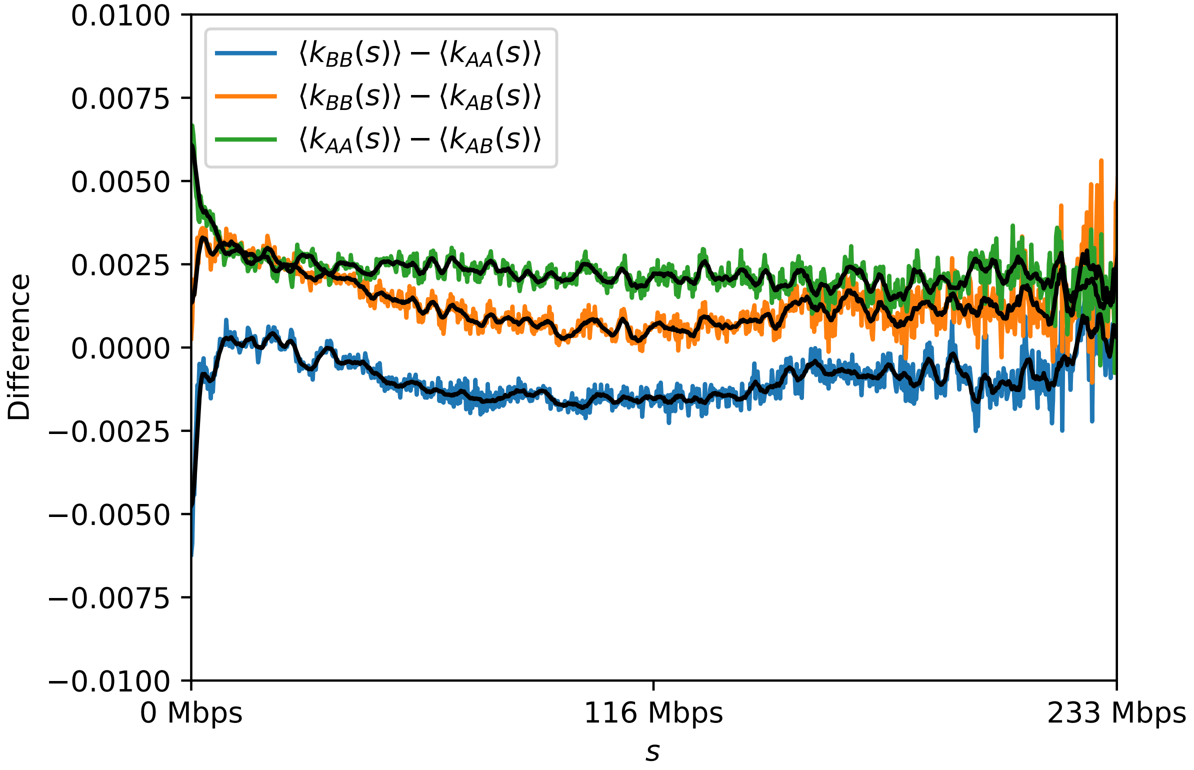

FIG. 7. $\langle k_{AA}(s) \rangle - \langle k_{AB}(s) \rangle$, $\langle k_{AA}(s) \rangle - \langle k_{BB}(s) \rangle$, and $\langle k_{BB}(s) \rangle - \langle k_{AB}(s) \rangle$ as a function of the genomic distance s. Black lines are the moving average over window size of $s = 2.6$Mbps

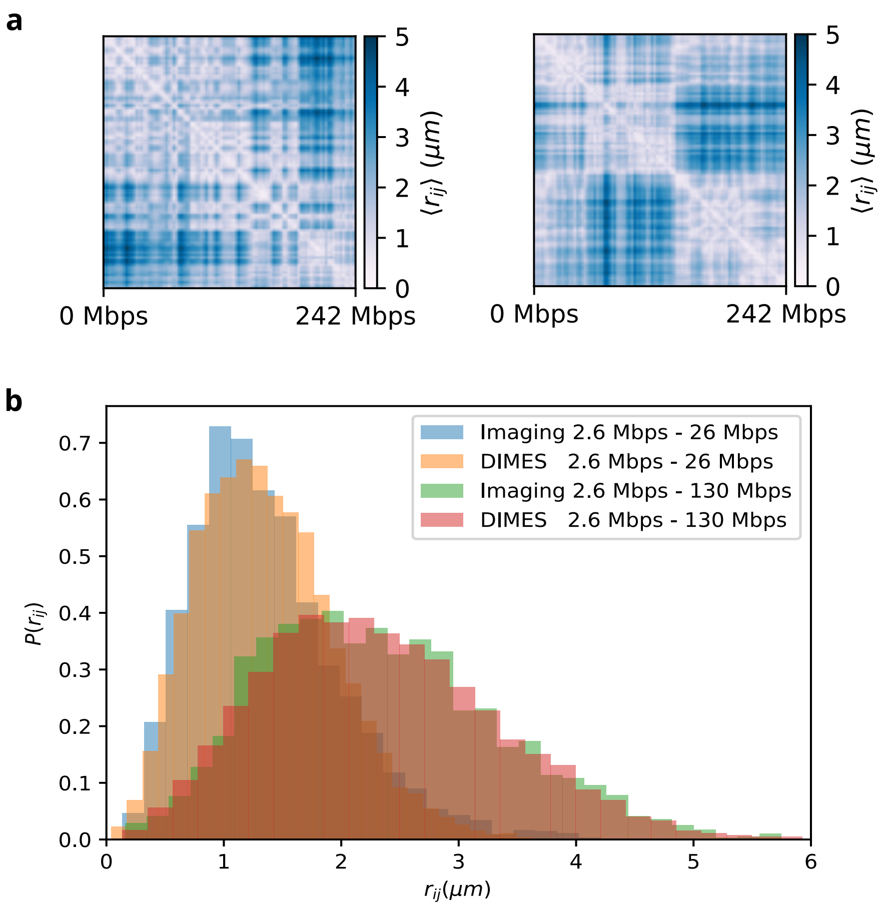

FIG. 8. **(a)** Experimental single-cell distance maps for Chr2 calculated from imaging data [6]. **(b)** Comparison of distributions of pairwise distances between imaging data and the prediction from DIMES. Pair 2.6 Mbps - 26 Mbps and pair 2.6 Mbps - 130 Mbps are shown.

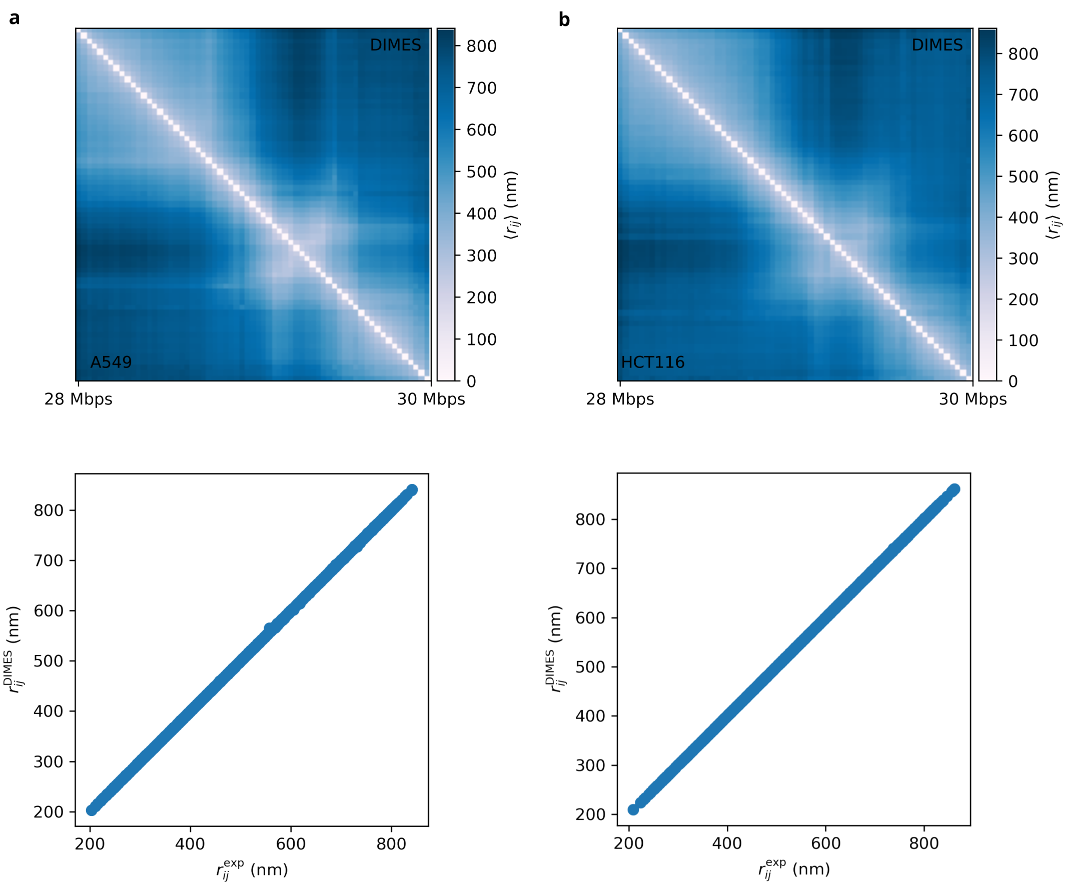

FIG. 9. Efficacy of DIMES in matching the targets generated using experimental imaging data. Comparison between the mean spatial distances computed from the reconstructed structures and the experimental data in cell lines: A549 (**a**), and HCT116 (**b**). The upper panel shows side-by-side comparisons of the distance matrices, and the lower panel displays the scatter plot between individual pairwise distances r_{ij}'s.

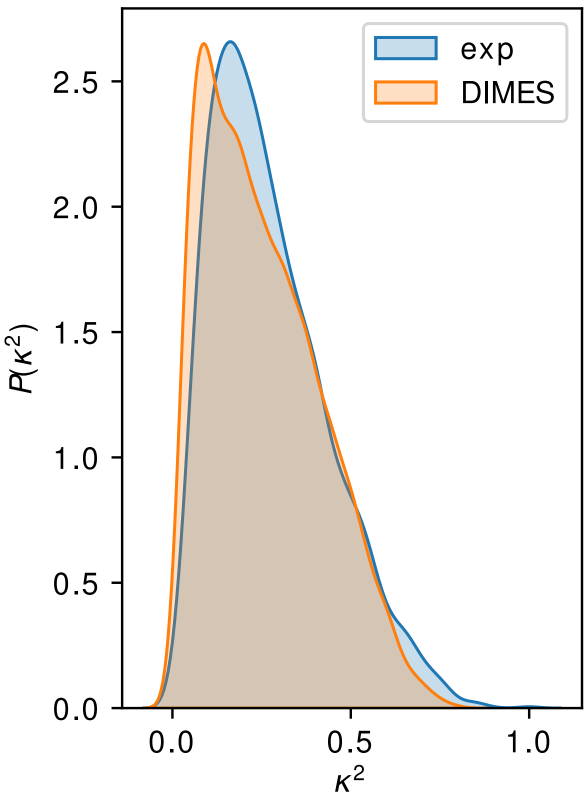

FIG. 10. Comparison of $P(\kappa^2)$ between experiment and the DIMES predictions. $P(\kappa^2)$ is the probability density distribution of the shape parameter κ^2 for the 28 Mbps - 30 Mbps region of Chr21.

SUPPLEMENTARY REFERENCES

[1] Agmon, N., Alhassid, Y. & Levine, R. An algorithm for finding the distribution of maximal entropy. *Journal of Computational Physics* **30**, 250–258 (1979).

[2] Mead, L. R. & Papanicolaou, N. Maximum entropy in the problem of moments. *Journal of Mathematical Physics* **25**, 2404–2417 (1984).

[3] Darroch, J. N. & Ratcliff, D. Generalized iterative scaling for log-linear models. *The annals of mathematical statistics* 1470–1480 (1972).

[4] Virtanen, P. *et al.* SciPy 1.0: Fundamental Algorithms for Scientific Computing in Python. *Nature Methods* **17**, 261–272 (2020).

[5] Bintu, B. *et al.* Super-resolution chromatin tracing reveals domains and cooperative interactions in single cells. *Science* **362**, eaau1783 (2018).

[6] Su, J.-H., Zheng, P., Kinrot, S. S., Bintu, B. & Zhuang, X. Genome-scale imaging of the 3D organization and transcriptional activity of chromatin. *Cell* **182**, 1641–1659.e26 (2020).
