# Supplementary material for: A maximum-entropy model to predict 3D structural ensembles of chromatin from pairwise distances with applications to interphase chromosomes and structural variants
Source: Nat Commun. 2023 Mar 1;14:1150. doi: 10.1038/s41467-023-36412-4 (PMC9974990; doi:10.1038/s41467-023-36412-4)
Supplement: Supplementary file 3 — Reporting Summary [file 41467_2023_36412_MOESM3_ESM.pdf]

nature portfolio

Corresponding author(s): Dave Thirumalai

Last updated by author(s): Jan 13, 2022

Reporting Summary

Nature Portfolio wishes to improve the reproducibility of the work that we publish. This form provides structure for consistency and transparency in reporting. For further information on Nature Portfolio policies, see our Editorial Policies and the Editorial Policy Checklist.

Statistics

For all statistical analyses, confirm that the following items are present in the figure legend, table legend, main text, or Methods section.

n/a	Confirmed	
☒	☐	The exact sample size (*n*) for each experimental group/condition, given as a discrete number and unit of measurement
☒	☐	A statement on whether measurements were taken from distinct samples or whether the same sample was measured repeatedly
☒	☐	The statistical test(s) used AND whether they are one- or two-sided<br>*Only common tests should be described solely by name; describe more complex techniques in the Methods section.*
☒	☐	A description of all covariates tested
☒	☐	A description of any assumptions or corrections, such as tests of normality and adjustment for multiple comparisons
☒	☐	A full description of the statistical parameters including central tendency (e.g. means) or other basic estimates (e.g. regression coefficient) AND variation (e.g. standard deviation) or associated estimates of uncertainty (e.g. confidence intervals)
☒	☐	For null hypothesis testing, the test statistic (e.g. *F*, *t*, *r*) with confidence intervals, effect sizes, degrees of freedom and *P* value noted<br>*Give P values as exact values whenever suitable.*
☒	☐	For Bayesian analysis, information on the choice of priors and Markov chain Monte Carlo settings
☒	☐	For hierarchical and complex designs, identification of the appropriate level for tests and full reporting of outcomes
☒	☐	Estimates of effect sizes (e.g. Cohen's *d*, Pearson's *r*), indicating how they were calculated

Our web collection on statistics for biologists contains articles on many of the points above.

Software and code

Policy information about availability of computer code

Data collection	The DIMES method described in the study is implemented using Python 3. The code for DIMES method and its detailed user instruction can be accessed at the Github repository https://github.com/anyuzx/HIPPS-DIMES. The Python packages used in the code are Numpy, Scipy, Pandas, Click, Cooler, tqdm
Data analysis	The data analysis is performed using Python 3.9 in Jupyter Lab. The Python packages used in data analysis are Scipy, Numpy, Pandas

For manuscripts utilizing custom algorithms or software that are central to the research but not yet described in published literature, software must be made available to editors and reviewers. We strongly encourage code deposition in a community repository (e.g. GitHub). See the Nature Portfolio guidelines for submitting code & software for further information.

Data

Policy information about availability of data

All manuscripts must include a data availability statement. This statement should provide the following information, where applicable:

- Accession codes, unique identifiers, or web links for publicly available datasets
- A description of any restrictions on data availability
- For clinical datasets or third party data, please ensure that the statement adheres to our policy

All relevant data supporting the findings of this study are available within the article and its Supplementary Information. The Multiplexed FISH imaging data used in this study are publicly available from the GitHub repository at https://github.com/BogdanBintu/ ChromatinImaging and Zenodo repository at https://zenodo.org/

record/3928890#.Yizd1xDMKFF. The Hi-C data used in this study is publicly avail- able from GEO database under accession num- ber GSE92294 [https://www.ncbi.nlm.nih.gov/ geo/query/acc.cgi?acc=GSE92294] and GSE63525 [https://www.ncbi.nlm.nih.gov/geo/query/acc. cgi?acc=GSE63525].

Human research participants

Policy information about studies involving human research participants and Sex and Gender in Research.

Reporting on sex and gender	n/a
Population characteristics	n/a
Recruitment	n/a
Ethics oversight	n/a

Note that full information on the approval of the study protocol must also be provided in the manuscript.

Field-specific reporting

Please select the one below that is the best fit for your research. If you are not sure, read the appropriate sections before making your selection.

☒ Life sciences ☐ Behavioural & social sciences ☐ Ecological, evolutionary & environmental sciences

For a reference copy of the document with all sections, see nature.com/documents/nr-reporting-summary-flat.pdf

Life sciences study design

All studies must disclose on these points even when the disclosure is negative.

Sample size	When applicable, we use DIMES to randomly and independently sample the genome structures 10000 times to obtain an ensemble of structures. 10000 independently sampled structures are sufficient to obtain the average quantity that is in agreement with experimental constraints
Data exclusions	No data is excluded in this study
Replication	This is computational study. No replication experiment is performed
Randomization	Not applicable to computational study
Blinding	Not applicable to computational study

Reporting for specific materials, systems and methods

We require information from authors about some types of materials, experimental systems and methods used in many studies. Here, indicate whether each material, system or method listed is relevant to your study. If you are not sure if a list item applies to your research, read the appropriate section before selecting a response.

Materials & experimental systems

n/a	Involved in the study
☒	☐ Antibodies
☒	☐ Eukaryotic cell lines
☒	☐ Palaeontology and archaeology
☒	☐ Animals and other organisms
☒	☐ Clinical data
☒	☐ Dual use research of concern

Methods

n/a	Involved in the study
☒	☐ ChIP-seq
☒	☐ Flow cytometry
☒	☐ MRI-based neuroimaging
